# Boron Oxide B_5_O_6_^−^ Cluster as a Boronyl-Based Inorganic Analog of Phenolate Anion

**DOI:** 10.3389/fchem.2022.868782

**Published:** 2022-04-08

**Authors:** Shu-Juan Gao, Jin-Chang Guo, Hua-Jin Zhai

**Affiliations:** ^1^ Nanocluster Laboratory, Institute of Molecular Science, Shanxi University, Taiyuan, China; ^2^ Department of Chemistry and Chemical Engineering, Lvliang University, Lvliang, China

**Keywords:** boron oxide clusters, boronyl, heteroatomic hexagonal B_3_O_3_ ring, chemical bonding, superhalogen anions

## Abstract

Boron oxide clusters have structural richness and exotic chemical bonding. We report a quantum chemical study on the binary B_5_O_6_^−^ cluster, which is relatively oxygen-rich. A global structural search reveals planar *C*_2*v*_ (^1^A_1_) geometry as the global minimum structure, featuring a heteroatomic hexagonal B_3_O_3_ ring as its core. The three unsaturated B sites are terminated by two boronyl (BO) groups and an O^−^ ligand. The B_5_O_6_^−^ cluster can be faithfully formulated as B_3_O_3_(BO)_2_O^−^. This structure is in stark contrast to that of its predecessors, *C*_*s*_ B_5_O_5_^−^ and *T*_*d*_ B_5_O_4_^−^, both of which have a tetrahedral B center. Thus, there exists a major structural transformation in B_5_O_*n*_^−^ series upon oxidation, indicating intriguing competition between tetrahedral and heterocyclic structures. The chemical bonding analyses show weak 6π aromaticity in the B_5_O_6_^−^ cluster, rendering it a boronyl analog of phenolate anion (C_6_H_5_O^−^) or boronyl boroxine. The calculated vertical detachment energy of B_5_O_6_^−^ cluster is 5.26 eV at PBE0, which greatly surpasses the electron affinities of halogens (Cl: 3.61 eV), suggesting that the cluster belongs to superhalogen anions.

## Introduction

Boron is an electron-deficient element with the capacity to build strong covalent bonds with other elements. Owing to boron’s high affinity to oxygen, boron oxide clusters readily form, exhibiting usual structures and exotic chemical bonding (Doyle, 1988; Peiris et al., 1997; Zhai et al., 2007a; Zhai et al., 2007b; Drummond et al., 2007; Li et al., 2008; Shao et al., 2009; Tai and Nguyen, 2009; Yao et al., 2009; Braunschweig et al., 2010; Tai et al., 2010; Zhai et al., 2011; Guo et al., 2013; Li et al., 2013; Miao et al., 2013; Zhai et al., 2014a; Chen et al., 2014; Tian et al., 2015a; Tian et al., 2015b; Zhao et al., 2016; Li et al., 2018; Feng et al., 2019; Li et al., 2019). Elemental boron clusters are intrinsically electron-deficient themselves (Zhai et al., 2003; Alexandrova et al., 2006), and therefore boron oxide clusters are anticipated to be even more electron-deficient. In boron-rich oxide clusters, the boronyl (BO) group has recently emerged as an interesting inorganic ligand (Zhai et al., 2014a), which features a robust B≡O triple bond. In fact, the diatomic BO and BO^−^ species are isoelectronic to CN and CN^−^/CO, respectively. In addition, boronyl is a monovalent σ radical, thus leading to isolobal analogy between the BO and H ligands. As the oxidation proceeds, direct B−B bonding gradually diminishes. Consequently, heteroatomic B−O rings start to appear, which serve as the structural core of boron oxide clusters. Such a heteroatomic ring can be rhombic, pentagonal, hexagonal, or polycyclic, giving rise to a diversity of boron oxide cluster structures that mimic aromatic hydrocarbons (including polycyclic aromatic hydrocarbons, PAHs). Among these is the heterocyclic hexagonal B_3_O_3_ ring, whose relevant clusters are classified as inorganic benzenes. Typical examples are boroxine (B_3_O_3_H_3_) and, more recently, the boronyl boroxine B_3_O_3_(BO)_3_ cluster (Li et al., 2013).

It is interesting and invaluable to follow the oxidation process of a specific bare boron cluster, step by step. The effort should help precisely identify a variety of cluster structures and structural transformations. Along this line, the B_5_O_*n*_^−^ clusters are an intriguing and informative system, which have been extensively studied in the past years (Yao et al., 2009; Zhai et al., 2011; Tian et al., 2015b), (Chen et al., 2012; Chen et al., 2013), (Zhai et al., 2002), including a number of gas-phase spectroscopic works. The B_5_^−^ cluster assumes double-chain ribbon geometry with a “W” shape (Zhai et al., 2002), while the B_5_^+^ cluster is pentagonal (Alexandrova et al., 2006). The B_5_O_*n*_^−^ (*n* = 1–3) clusters are entirely dictated by boronyl groups, whose number increases steadily from 1 to 3, reducing the size of boron core from rhombic B_4_ to triangular B_3_ and then to dimer B_2_ (Zhai et al., 2011; Chen et al., 2012; Chen et al., 2013). The first one or two boronyl ligands are terminally attached to the boron core, whereas the third one occupies a bridging position. The coordination pattern is similar to a hydrogen ligand in boranes, demonstrating the BO/H isolobal analogy. It is noted that all B_5_O_*n*_^−^ (*n* = 0–3) clusters are perfectly planar. In the B_5_O_4_^−^ cluster (Yao et al., 2009), a tetrahedral geometry occurs, which is still governed by boronyl ligands, except that their number increases to four. It can be formulated as B(BO)_4_^−^, being isovalent to BH_4_^−^ or CH_4_. The B_5_O_5_^−^ cluster inherits the tetrahedral geometry of B_5_O_4_^−^ upon substitution of one boronyl terminal by a linear OBO unit (Tian et al., 2015b). What is next in the sequential oxidation process of bare B_5_ cluster? Would the tetrahedral geometry persist? What new type of structure would appear? These remain to be open questions in the field.

In the present work, we are motivated to address the abovementioned issues. We report on the structural, electronic, and chemical bonding properties of boron oxide B_5_O_6_^−^ cluster *via* computer global searches and quantum chemical calculations. It turns out that the global minimum (GM) structure of B_5_O_6_^−^ cluster features a heterocyclic B_3_O_3_ core, whose three unsaturated B sites are decorated by two BO ligands and one O^−^ unit. Herein, the abbreviation “GM” refers to a structure that is lowest in energy on the potential energy surface of a specific molecular system, which is routinely used in physical chemistry or cluster literature. The hexagonal B_3_O_3_ core is stabilized by a moderately delocalized 6π system, thus rendering the B_5_O_6_^−^ cluster a boronyl-based analog of benzene, akin to boroxine or boronyl boroxine (Li et al., 2013). In terms of the overall chemical bonding pattern, it is proposed that the B_5_O_6_^−^ cluster closely mimics phenolate anion (C_6_H_5_O^−^). The relatively localized extra charge (O^−^) in B_5_O_6_^−^ cluster gives rise to high vertical detachment energy (VDE) beyond 5 eV, indicating that the cluster is a superhalogen anion (Gutsev and Boldyrev, 1984; Srivastava, 2022; Kandalam et al., 2015; A Technical Note).

## Methods

The GM structural searches for B_5_O_6_^−^ cluster were carried out at the PBE0/3-21G level using the coalescence kick (CK) algorithm (Saunders, 2004; Sergeeva et al., 2011). A total of 8000 stationary points were probed on its potential energy surface (4000 for singlet and triplet states each). The identified low-lying isomers were then fully reoptimized at the PBE0/6-311+G(d) level and their relative energies evaluated, including zero-point energy (ZPE) corrections. The vibrational frequencies were calculated at the same level to ensure that the reported structures are true minima on the potential energy surface.

Our further effort to assess the energetics for top candidate structures is as follows. First, the PBE0-D3/6-311+G(d) calculations were carried out to take into account dispersion correction. Second, comparative B3LYP-D3/6-311+G(d) calculations were carried out to check for consistency of density functionals; the PBE0 and B3LYP functionals have been widely considered to be complementary with each other. Third, single-point CCSD(T)/6-311+G(d) calculations (Purvis and Bartlett, 1982) were carried out on the basis of optimized PBE0-D3/6-311+G(d) geometries, which shall serve as a benchmark of the energetics data. All four sets of energetics data are highly consistent.

The NBO 6.0 program (Glendening et al., 2013) was used for the natural bond orbital (NBO) (Reed et al., 1988) analysis, which offers Wiberg bond indices (WBIs) and natural atomic charges. Chemical bonding was elucidated v*ia* canonical molecular orbital (CMO) analysis, adaptive natural density partitioning (AdNDP) (Zubarev and Boldyrev, 2008), and electron local functions (ELFs) (Silvi and Savin, 1994). To assess π aromaticity, nucleus-independent chemical shifts (NICSs) were also calculated (Schleyer et al., 1996), which were supplemented by the isochemical shielded surface (ICSS) analysis (Guo et al., 2020). The latter was performed using the Multiwfn program (Lu and Chen, 2012). The VDEs were calculated using the time-dependent PBE0 (TD-PBE0) (Bauernschmitt and Ahlrichs, 1996; Casida et al., 1998) and outer valence Green’s function (OVGF) (von Niessen et al., 1984; Zakrzewski and von Niessen, 1993; Zakrzewski and Ortiz, 1995) methods and at the single-point CCSD(T) level. All the electronic structure calculations were carried out using the Gaussian 09 package (Frisch, 2009).

## Results

### Global Minimum Structure and Alternative Low-Lying Isomers

**FIGURE 1:**
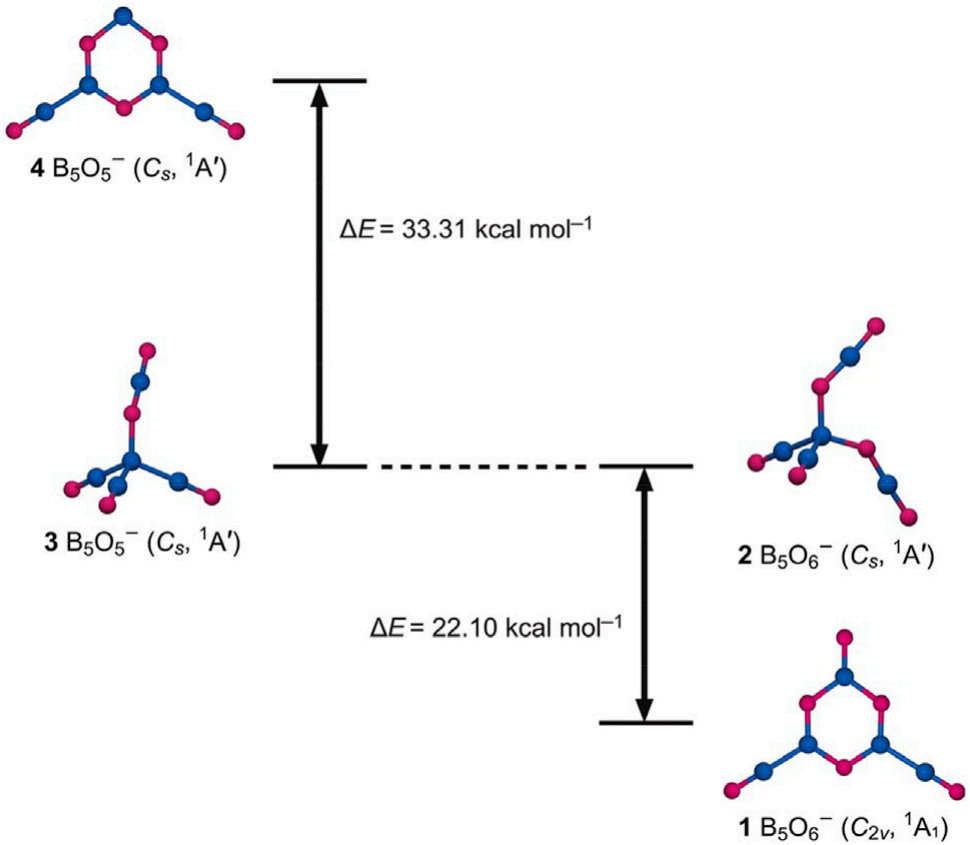
Global minimum (GM) structure of B_5_O_6_^−^ (1, *C*_2*v*_, ^1^A_1_) cluster and a representative 2 (*C*_*s*_, ^1^A′) isomer. They feature a heterocyclic hexagonal B_3_O_3_ ring and a tetrahedral B center, respectively. Relevant structures 3/4 of B_5_O_5_^−^ cluster (left panels) show a distinctly reversed energy order. The relative energies are shown in kcal mol^−1^ at the PBE0/6-311+G(d) level.

**FIGURE 2:**
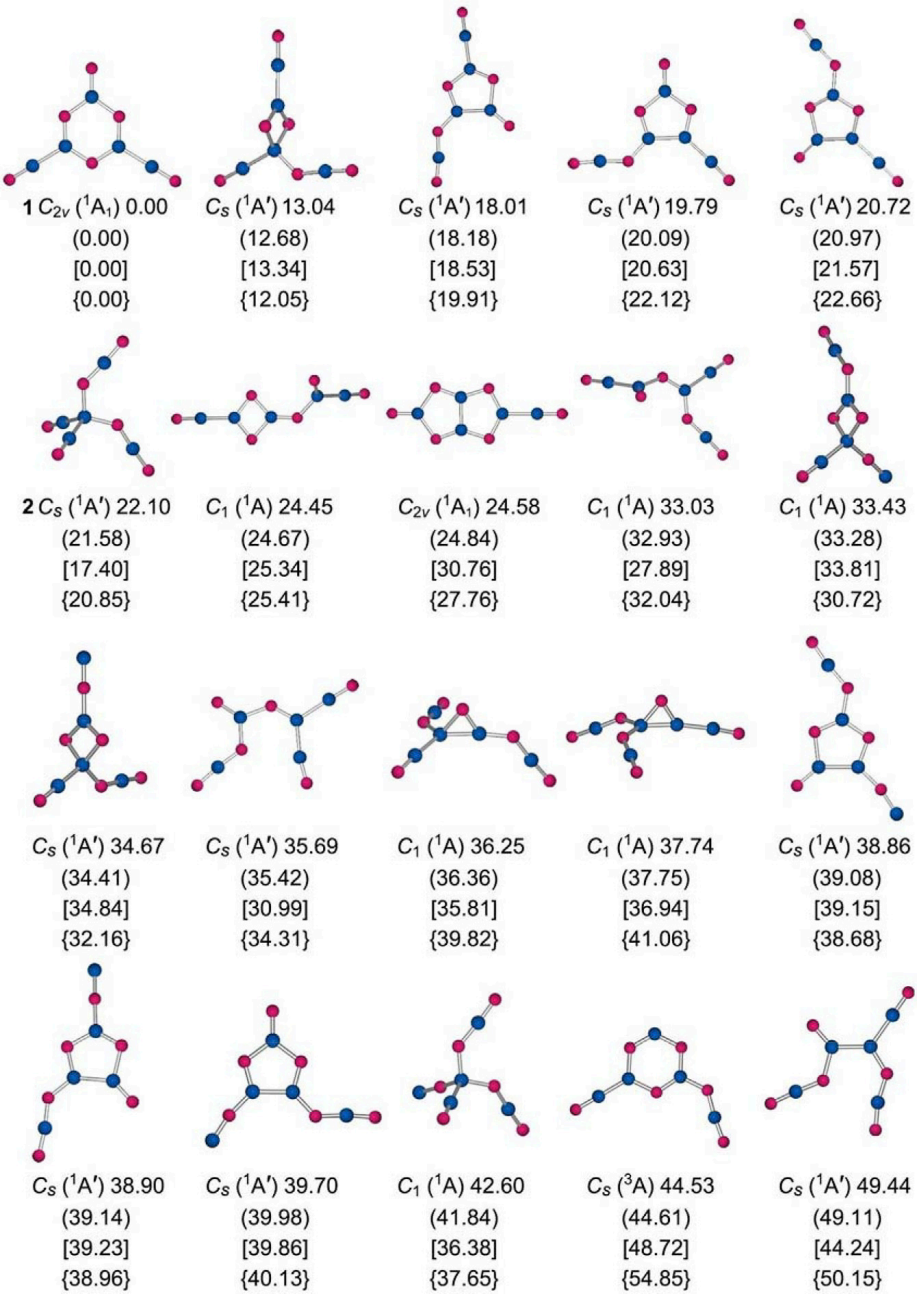
Alternative optimized low-lying isomeric structures of B_5_O_6_^−^ cluster at the PBE0/6-311+G(d) level along with their relative energies (in kcal mol^−1^), including zero-point energy (ZPE) corrections. The energetics data are also presented at the PBE0-D3/6-311+G(d) (in brackets, with ZPE corrections) and B3LYP-D3/6-311+G(d) (in square brackets, with ZPE corrections) levels, as well as at single-point CCSD(T)/6-311+G(d) level based on their PBE0-D3/6-311+G(d) geometries (in curly brackets).

Our computational structural searches lead to the GM structure 1 (*C*_2*v*_, ^1^A_1_) for anion B_5_O_6_^−^ cluster; see Figure 1. Alternative low-lying isomeric structures are presented in Figure 2 along with their relative energies at four levels of theory: PBE0/6-311+G(d), PBE0-D3/6-311+G(d), and B3LYP-D3/6-311+G(d) levels, as well as the single-point CCSD(T)/6-311+G(d) level on the basis of PBE0-D3/6-311+G(d) geometries. The four sets of independent energetics data are highly consistent (Figure 2), firmly establishing cluster 1 as the GM structure. It is relatively well-defined on the potential energy surface, being at least 12 kcal mol^−1^ more stable than any alternative geometry. It is noted that the PBE0 functional has been widely tested for boron clusters (Zhai et al., 2014b; Fagiani et al., 2017; Guo et al., 2017), which is a popular and reliable choice for boron-based systems.

Cluster 1 is closed-shell. The lowest-energy triplet structure is 54.85 kcal mol^−1^ higher in energy at the single-point CCSD(T) level (Figure 2). Among the low-lying isomers is a tetrahedral 2 (*C*_*s*_, ^1^A′) structure, which is 22.10 kcal mol^−1^ above GM cluster 1 at PBE0 [20.85 kcal mol^−1^ at single-point CCSD(T)]. Its geometry can be traced back to the GM structure 3 (*C*_*s*_, ^1^A′) of anion B_5_O_5_^−^ cluster (Figure 1) (Tian et al., 2015b), upon substitution of one terminal boronyl by an OBO unit. For the latter cluster, hexagonal isomer 4 (*C*_*s*_, ^1^A′) is substantially higher in energy than GM cluster 3, by as much as 33.31 kcal mol^−1^ at PBE0. Structures 1–4 have an intriguing energetics relationship; see Figure 1. A clear structural transition occurs from B_5_O_5_^−^ to B_5_O_6_^−^. The optimized Cartesian coordinates of structures 1 and 2 are presented in Supplementary Table S1 (ESI†).

### Bond Distances, Wiberg Bond Indices, and Natural Atomic Charges

**FIGURE 3:**
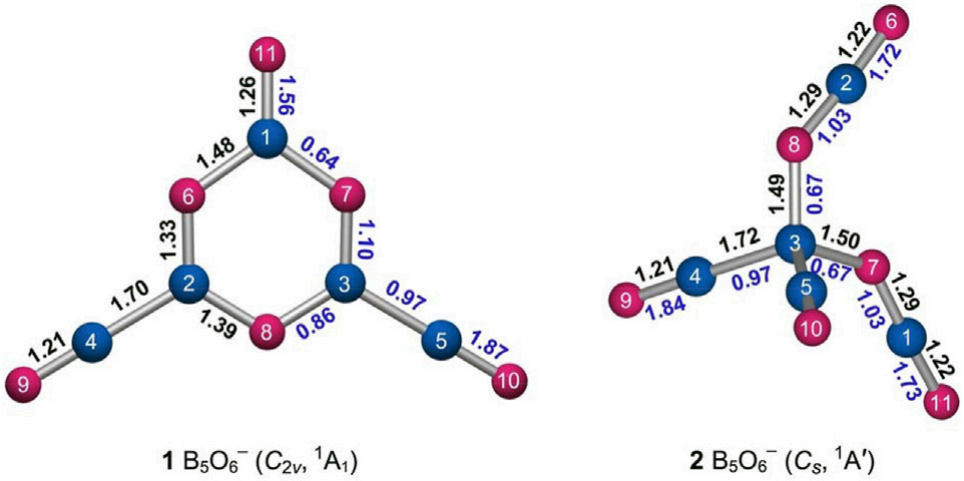
Optimized bond distances (in Å; black color) of GM structure 1 and isomer 2 of the B_5_O_6_^−^ cluster at the PBE0/6-311+G(d) level. Also shown are their Wiberg bond indices (WBIs; blue color) from the natural bond orbital (NBO) analysis. The B atoms are illustrated in blue and O in red.

As shown in Figure 3, the perfectly planar GM B_5_O_6_^−^ (1, *C*_2*v*_, ^1^A_1_) cluster contains a heteroatomic hexagonal B_3_O_3_ ring, two-terminal BO ligands, and one terminal O unit. The BO or O units are attached to three B sites on the periphery. The structure is relatively straightforward to elucidate based on interatomic distances. Typical triple B≡O, double B=O, single B−O, and single B−B bonds are around 1.21, 1.28, 1.37, and 1.66 Å, respectively (Yao et al., 2009; Zhai et al., 2014a; Tian et al., 2015b; Pyykkö and Atsumi, 2009). The bond distances for BO links in hexagonal ring of cluster 1 are slightly uneven (1.33–1.48 Å; Figure 3, left panel) due to asymmetric coordination of B sites. Their average value is in line with single B−O bonds. The terminal BO groups (1.21 Å) are assigned as boronyls with triple B≡O bonds. In contrast, the upper BO unit (1.26 Å) is close to a double B=O bond.

**FIGURE 4:**
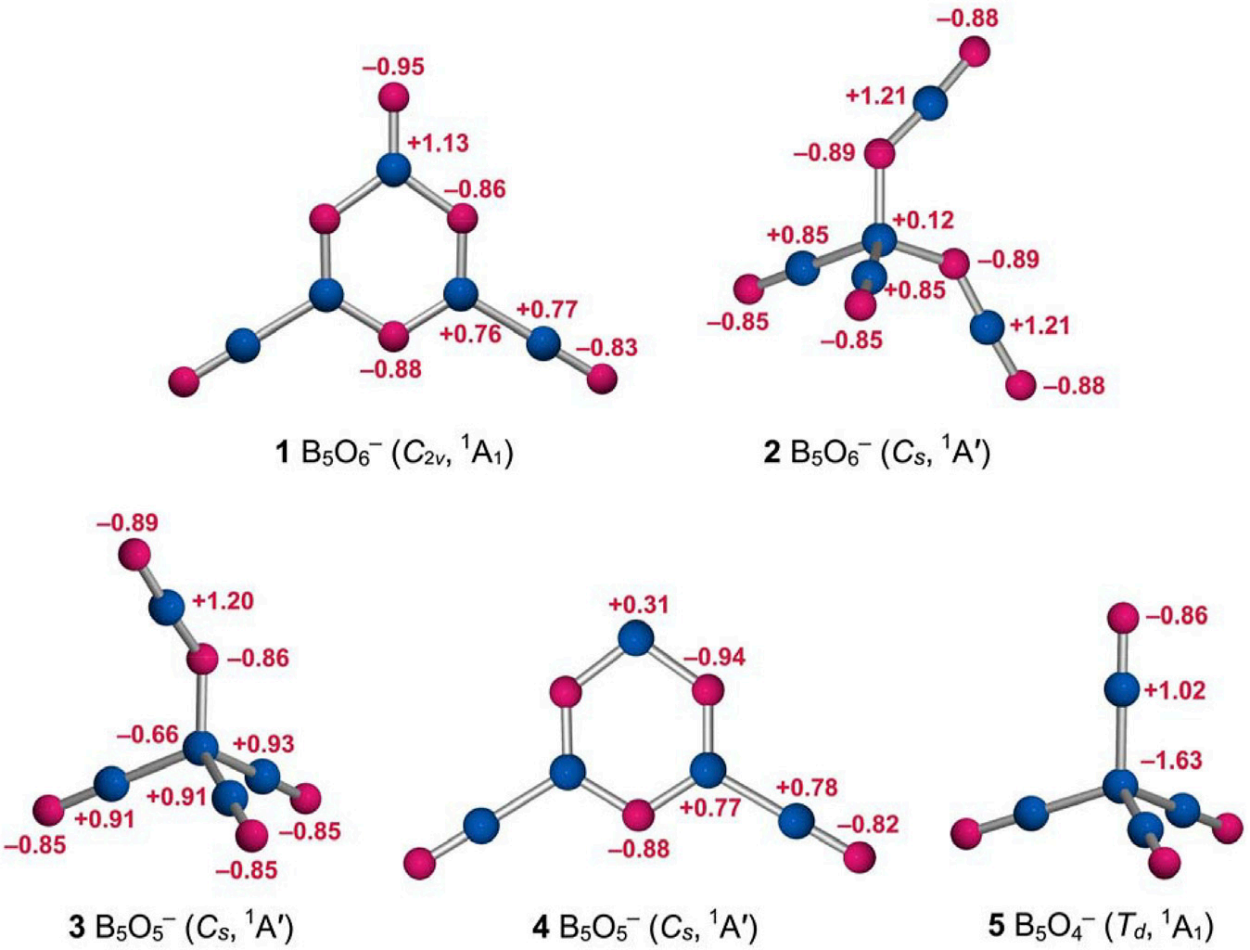
Calculated natural atomic charges (in |e|) for structures 1 and 2 of the B_5_O_6_^−^ cluster from the NBO analysis at PBE0/6-311+G(d), as compared to those of B_5_O_5_^−^ (3, *C*_*s*_, ^1^A′), B_5_O_5_^−^ (4, *C*_*s*_, ^1^A′), and B_5_O_4_^−^ (5, *T*_*d*_, ^1^A_1_) clusters. The B atoms are shown in blue and O in red.

The calculated WBIs generally confirm the abovementioned assignments (Figure 3, left panel). The inner BO links have WBI values of 0.64–1.10, in line with (uneven) single bonds. The boronyl groups have WBIs of 1.87 owing to their polar nature (Zhai et al., 2014a). The upper BO unit has a smaller WBI value of 1.56, in line with a double bond. The calculated natural atomic charges for cluster 1 are shown in Figure 4. There are moderate intramolecular B−O charge transfers within the B_3_O_3_ core, within boronyl groups, and in between the core and terminals. The charge transfers are relatively local processes, suggesting polar and yet covalent BO chemical bonding.

Likewise, the isomeric tetrahedral B_5_O_6_^−^ (2, *C*_*s*_, ^1^A′) structure can be easily understood (Figures 3, 4). It consists of a central B site, two BO groups, and two OBO groups. The components are held together in a tetrahedral fashion. Approximately, in anion cluster, the B center forms four B−B or B−O single bonds with terminal ligands, whose B−B (1.72 Å) and B−O (1.49–1.50 Å) links are somewhat elongated with respect to the abovementioned reference values for single bonds. However, their WBIs are 0.97 and 0.67, respectively, and even the latter seems reasonable (for a polar bond). Such a bonding situation around the B center is less than ideal, which is partly the reason why this structure is a higher-energy isomer (*vide infra*). For the terminal ligands, two BO groups (1.21 Å) are boronyls with WBIs of 1.84. The two OBO units are asymmetric in terms of B−O distances: 1.22 versus 1.29 Å. Their average is close to a double B=O bond. The calculated natural atomic charges of cluster 2 are presented in Figure 4. The central B site is practically neutral (+0.12 |e|) despite the fact that other B atoms each carry a larger positive charge (+0.85 and +1.21 |e|). This situation again hints that the central B site struggles severely in order to maintain four-fold bonding.

To aid the understanding of GM *C*_2*v*_ B_5_O_6_^−^ (1) and isomeric *C*_*s*_ B_5_O_6_^−^ (2) clusters, three prior species (Yao et al., 2009; Tian et al., 2015b) are also analyzed: tetrahedral GM *C*_*s*_ B_5_O_5_^−^ (3), hexagonal isomeric *C*_*s*_ B_5_O_5_^−^ (4), and tetrahedral GM *T*_*d*_ B_5_O_4_^−^ (5). Their calculated bond distances and WBIs (Supplementary Figure S1, ESI†) and natural atomic charges (Figure 4) are well behaved, providing support to our assessment with regard to clusters 1 and 2 (as described above).

### Superhalogen Anion

**FIGURE 5:**
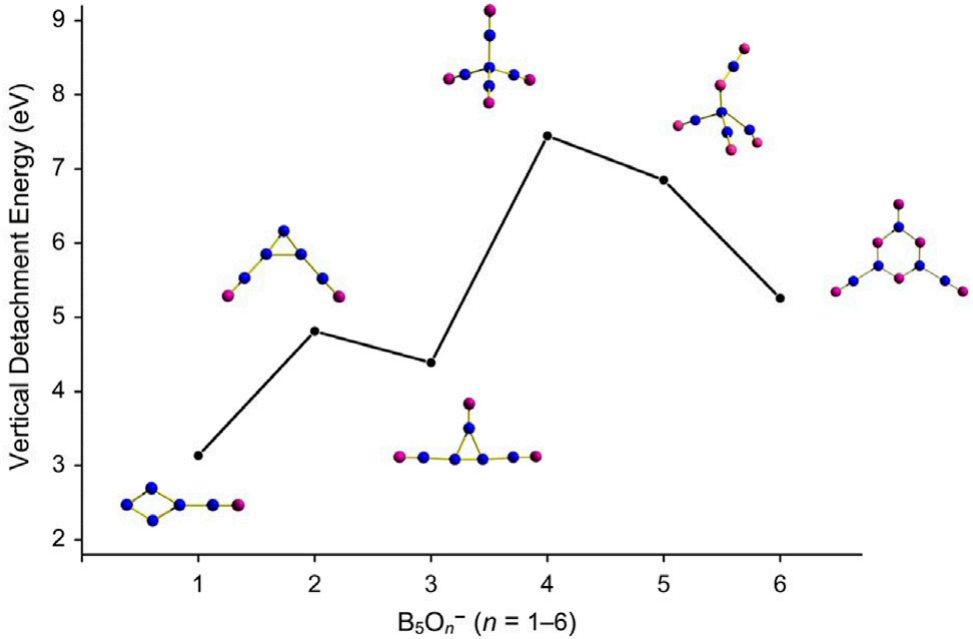
Evolution of ground-state vertical detachment energies (VDEs) of B_5_O_*n*_^−^ (*n* = 1–6) series calculated at the PBE0/6-311+G(d) level.

The electronic properties of GM B_5_O_6_^−^ (1) cluster are predicted here to aid its future experimental characterization in the gas phase. The calculated ground-state VDE of B_5_O_6_^−^ (1) cluster and those of a few relevant species are presented in Supplementary Table S2 (ESI†) at three levels of theory: PBE0, single-point CCSD(T), and OVGF. Using the experimentally known B_4_O_3_^−^ cluster (Zhai et al., 2007a) as a calibration, it is shown that the PBE0 and single-point CCSD(T) data are superior to those of OVGF; see a note in Supplementary Table S2 (ESI†). The VDE of GM B_5_O_6_^−^ (1) cluster amounts to 5.26 eV at PBE0 and 5.14 eV at CCSD(T), which is quite high, markedly surpassing the electron affinities of halogens (Cl: 3.61 eV). The ground-state VDEs of the whole B_5_O_*n*_^−^ (*n* = 1–6) series at PBE0/6-311+G(d) are plotted in Figure 5, which show an abrupt increase between *n* = 3 and 4. All the B_5_O_*n*_^−^ (*n* = 4–6) species have high VDEs. Based on this observation, the GM B_5_O_6_^−^ (1) cluster clearly belongs to the class of species called superhalogen anions. (Gutsev and Boldyrev, 1984; Srivastava, 2022; Kandalam et al., 2015; A Technical Note)

**FIGURE 6:**
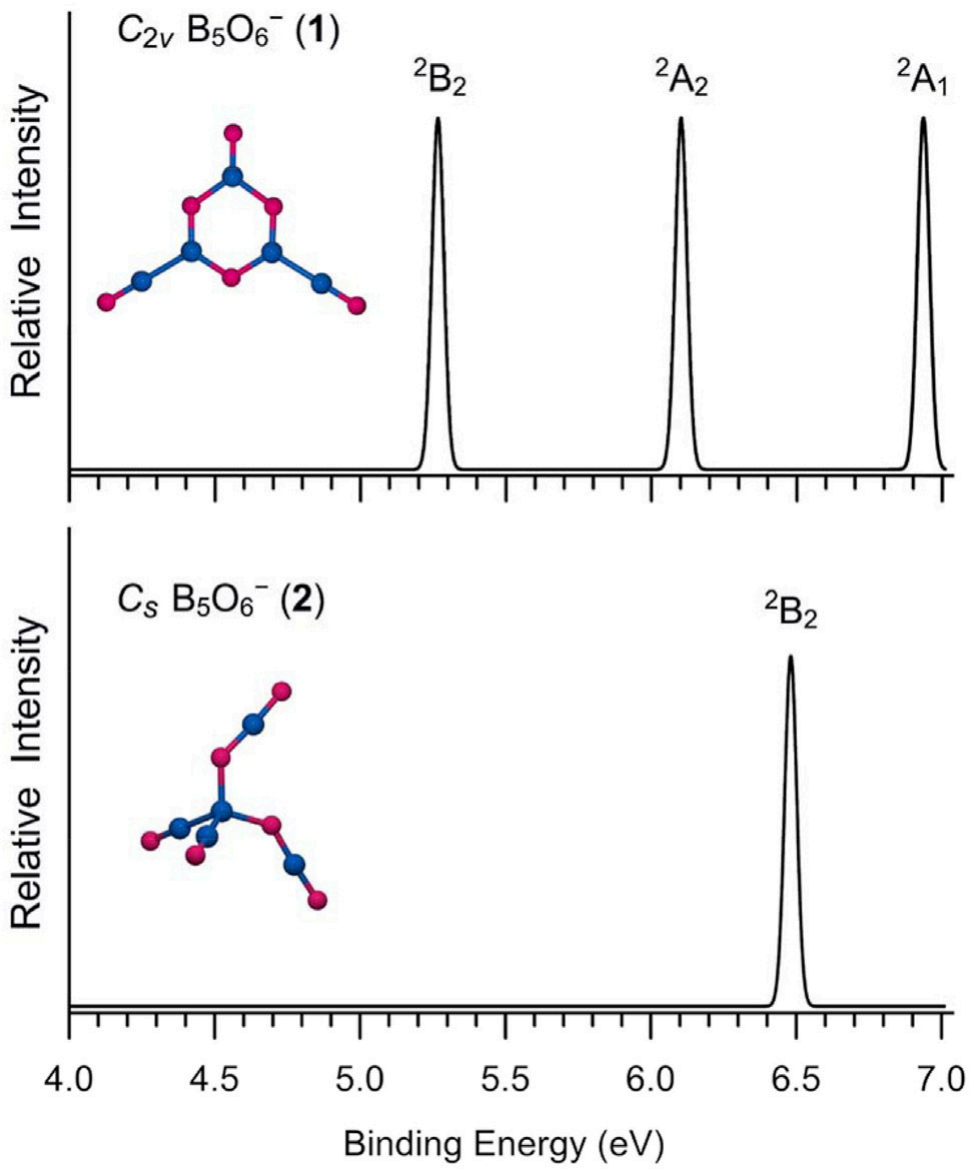
Simulated photoelectron spectra of anionic B_5_O_6_^−^ (1) and B_5_O_6_^−^ (2) clusters at the time-dependent PBE0/6-311+G(d) (TD-PBE0) level.

The simulated photoelectron spectrum of GM B_5_O_6_^−^ (1) cluster is presented in Figure 6, according to the TD-PBE0 calculation, which has three well-separated bands in the 5–7 eV binding energy regime. For comparison, the simulation is also carried out for isomeric structure 2 with a higher ground-state band (Figure 6B). A nature implication is that for their corresponding neutral species, the hexagonal structure has an even greater advantage in terms of energetics (over tetrahedral structure). Indeed, our PBE0 calculations show that the hexagonal neutral B_5_O_6_ cluster is 54.59 kcal mol^−1^ below its tetrahedral counterpart, as compared to a relative energy of 22.10 kcal mol^−1^ between anionic 1/2 structures at the same level (Figure 1). The two neutral structures are analyzed in Supplementary Figures S2, S3 (ESI†). For the hexagonal anion/neutral system, the extra charge goes primarily to the upper O terminal (by as much as 0.66 |e|), which further justifies the assessment of an O^−^ terminal for GM B_5_O_6_^−^ (1) cluster, as well as its exact analogy to phenolate anion (*vide infra*). In contrast, the extra charge in the tetrahedral system smears over the entire cluster (with the central B site gaining 0.34 |e| only), thus leading to an even higher ground-state VDE.

## Discussion

### Heterocyclic Hexagonal Global-Minimum *C*_2*v*_ B_5_O_6_^−^ Cluster: A Boronyl-Based Inorganic Analog of Phenolate Anion

The GM *C*_2*v*_ B_5_O_6_^−^ (1) cluster marks the exact onset of a heteroatomic hexagonal B_3_O_3_ ring along the whole B_5_O_*n*_^−^ (*n* = 0–6) series (Zhai et al., 2002; Yao et al., 2009; Zhai et al., 2011; Chen et al., 2012; Chen et al., 2013; Tian et al., 2015b). A simple valence electron counting suggests that the available number of electrons for direct B−B bonding diminishes gradually from 16 electrons in B_5_^−^ down to 4 in B_5_O_6_^−^, two electrons for each additional O atom. Indeed, both GM B_5_O_6_^−^ cluster 1 and isomeric structure 2 have four electrons for direct B−B bonding, that is, two B−B single bonds (see Figure 1).

**FIGURE 7:**
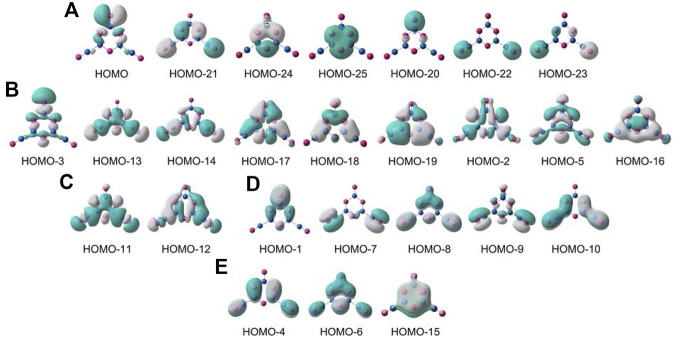
Pictures of occupied canonical molecular orbitals (CMOs) of B_5_O_6_^−^ (1) cluster. **(A)** Seven lone pairs for the O atoms, including two for the upper O site. **(B)** Nine σ CMOs for Lewis-type skeleton and terminal B−O σ bonds. **(C)** Two σ CMOs for Lewis-type B−B σ single bonds. **(D)** Five CMOs for both in-plane and out-of-plane π bonds relevant to terminal B−O units. **(E)** Three delocalized π CMOs over the B_3_O_3_ ring.

To fully understand GM B_5_O_6_^−^ (1) cluster, it is essential to conduct an in-depth analysis of chemical bonding. We choose to focus on the CMO analysis, which is fundamental for a molecular system. Cluster 1 is a closed-shell structure with 52 valence electrons. The 26 occupied CMOs are presented in Figure 7, which are sorted into five subsets according to their components of atomic orbitals (AOs). The seven CMOs in subset (a) are classified as O lone-pairs. Among them, HOMO−21/HOMO−24/HOMO−25 are typical bonding/nonbonding/antibonding combination of O 2s AOs from three O sites in the hexagonal ring owing to pseudo-three-fold symmetry of the cluster. According to the CMO building principles, they are readily recombined as three O 2s lone pairs. HOMO−20 is an O 2s lone-pair on the upper O site, whereas HOMO−22/HOMO−23 recombine as two O 2s lone-pairs on terminal boronyls. The HOMO approximately represents an O 2p lone-pair on the upper O site with 88% contribution from tangential O 2p AO. Thus, the upper O site has two lone pairs, and the remaining O sites each have one lone pair.

In subset (b), nine B−O σ single bonds are presented. Specifically, HOMO−3 is a σ bond for terminal B−O unit at the top. HOMO−13/HOMO−14 recombine as two σ bonds on the bottom boronyl groups. These are primarily contributed by radial O 2p AOs from three terminal O sites. The next six CMOs are responsible for skeleton B−O σ bonds along the hexagonal ring, which are evenly contributed by the B/O sites: radial O 2p AOs versus tangential B 2p AOs.

The CMOs in subset (c) are two B−B σ bonds, which link hexagonal rings and two boronyl ligands. The subset (d) shows the terminal B−O π bonds, including two in-plane π bonds on boronyls. Overall, the three-terminal B−O links each have one σ bond (Figure 7B) and one out-of-plane π bond (Figure 7D). Furthermore, the boronyl groups each have one in-plane π bond. The boronyls show triple bonds, whereas the upper B−O terminal has a double bond.

**FIGURE 8:**
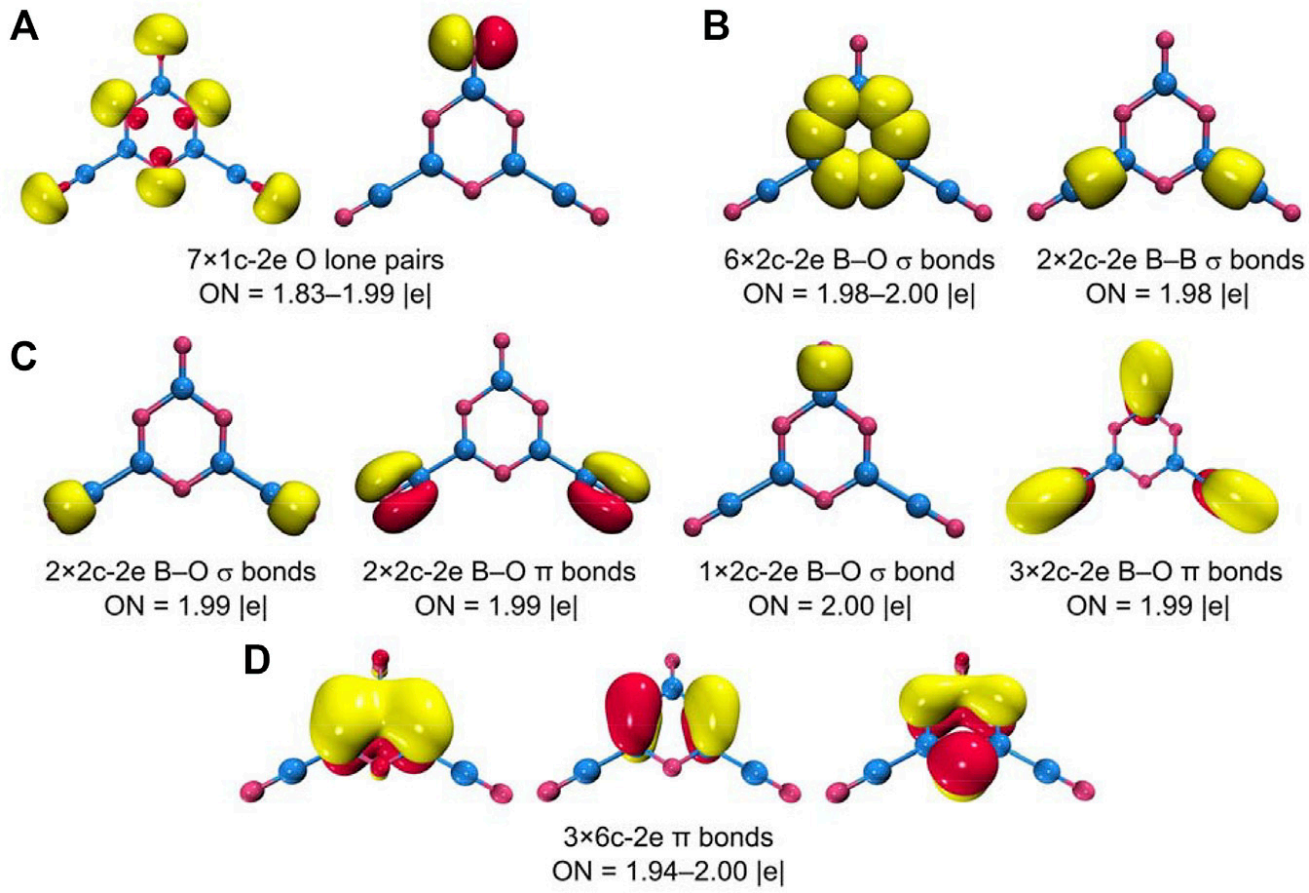
Chemical bonding pattern of B_5_O_6_^−^ (1) cluster according to the adaptive natural density partitioning (AdNDP) analysis. The occupation numbers (ONs) are indicated.

All the abovementioned CMOs are Lewis-type bonding elements: seven O 2s/2p lone-pairs, six B−O σ single bonds along the hexagon, two B−B σ bonds, one B=O double bond for the upper terminal, and two B≡O triple bonds for boronyls. These Lewis elements collectively consume 23 pairs of electrons, leaving the remaining six electrons for a π framework over the hexagonal ring (Figure 7E). Of the three π CMOs, HOMO−4 recombines partially with HOMO−10 to get “purified,” which has a B versus O ratio of roughly 1/11 in the hexagon. HOMO−6 has a ratio of 1/11 for B/O contributions. For HOMO−15, the ratio of B/O contributions in the ring boosts greatly to about 1/1. Overall, the B components in the π framework cannot be ignored, and the π sextet should be viewed as six-centered (at least formally). This π pattern is closely similar to that in boroxine and boronyl boroxine (Li et al., 2013), thus rendering GM B_5_O_6_^−^ (1) cluster a new member of the “inorganic benzene” family. The 6π electron-counting conforms to the (4*n* + 2) Hückel rule for aromaticity. The bonding picture is elegantly borne out from the AdNDP analysis (Zubarev and Boldyrev, 2008), as shown in Figure 8. All the occupation numbers (ONs) are close to ideal.

Based on the bonding picture, we should propose that GM B_5_O_6_^−^ (1) cluster is an exact boronyl-based analog of phenolate anion. They share the same characteristic structural and bonding features. First, they both have a hexagonal core: heteroatomic B_3_O_3_ ring versus C_6_ ring. The two kinds of rings are actually isoelectronic in terms of hexagonal bonding, once lone-pairs or terminal Lewis-type bonds are accounted for. Indeed, each ring consumes 18 electrons for chemical bonding within the ring. Second, both species possess a π sextet. Third, both species have an O^−^ terminal, which is attached to the hexagon *via* a double bond (B=O^−^ versus C=O^−^). It is appropriate to state that the current finding of chemical analogy between GM B_5_O_6_^−^ (1) cluster and phenolate anion parallels that between boronyl boroxine (Li et al., 2013) and benzene.

We shall only briefly mention the tetrahedral isomeric cluster B_5_O_6_^−^ (2). Its structural characters suggest a relatively classical cluster between a formal B^−^ center and four ligands (two boronyls versus two OBO units), *via* single bonds in a tetrahedral fashion. The chemical bonding within an OBO unit is presented in Supplementary Figure S4 (ESI†). Here, HOMO−16 and HOMO form a three-center, four-electron (3c-4e) π bond in the vicinity of an OBO unit, that is, “π hyperbond,” which is in a bonding/nonbonding combination due to its three-center nature. The upper CMO has relatively minor bonding or antibonding effect. Thus, a 3c-4e π bond is, in effect, equivalent to a 3c-2e bond or two B−O half bonds. There is a second 3c-4e π bond (HOMO−17 and HOMO−1) on the same OBO unit, which offers two B−O half bonds in the perpendicular direction. In short, the four CMOs in Supplementary Figure S4A (ESI†) can be collectively viewed as two B−O single bonds, similar to the prior tetrahedral *C*_*s*_ B_5_O_5_^−^ (3) cluster (Tian et al., 2015b) (see Supplementary Figure S4B, ESI†). An OBO unit in cluster 2 also has two B−O σ bonds (not shown). The same ideal works for the other OBO ligand. Overall, the OBO ligands can be formulated as O=B=O, featuring double bonds.

### Weak 6π Aromaticity in *C*_2*v*_ B_5_O_6_^−^ Cluster: Comparison With a Series of Relevant Species

**FIGURE 9:**
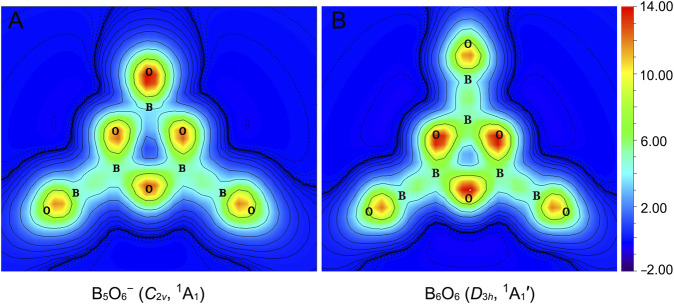
Color-filled maps of iso-chemical shielding surfaces (ICSSs) in the z-direction, ICSS_zz_ (in ppm), for **(A)** B_5_O_6_^−^ (1) and **(B)** boronyl boroxine *D*_3*h*_ B_6_O_6_ clusters. Positive ICSS_zz_ values indicate aromaticity. The calculations are carried out on a plane that is 1.0 Å above the molecular plane.

The π sextet in GM B_5_O_6_^−^ (1) cluster stems primarily from three O 2p_z_ lone-pairs in the hexagonal ring, taking advantage of the empty B 2p_z_ AOs from three neighboring B sites for six-centered π delocalization. Consequently, π aromaticity in cluster 1 is expected to be moderate only, despite its ideal 6π electron-counting. To quantitatively probe π aromaticity in cluster 1, we have calculated the color-filled maps of ICSS in the z-direction, that is, ICSS_zz_ (in ppm) (Guo et al., 2020), as shown in Figure 9A at 1.0 Å above the molecular plane. It noted that a positive ICSS_zz_ value indicates aromaticity. It turns out that the shielding effect at 1.0 Å above the ring center of cluster 1 is weak and only at a larger distance (such as 1.5 or 2.0 Å) does the shielding become apparent. Specifically, the calculated NICS_zz_ values for cluster 1 are +2.08 (likely due to disturbance from O lone-pairs), −3.24, and −4.02 ppm at 1.0, 1.5, and 2.0 Å, respectively, a trend in line with weak π aromaticity. For comparison, the corresponding NICS_zz_ values for boronyl boroxine are −1.97, −6.07, and −5.73 ppm, respectively, indicating slightly stronger π aromaticity.

Alternatively, we can also compare the extent of π aromaticity of GM B_5_O_6_^−^ (1) cluster and relevant species using the ELF data. (Silvi and Savin, 1994), (Wang et al., 2017) The π bifurcation value, ELF_π_, of GM B_5_O_6_^−^ (1) cluster is compared with those of boroxine *D*_3*h*_ B_3_O_3_H_3_, boronyl boroxine *D*_3*h*_ B_6_O_6_, and benzene *D*_6*h*_ C_6_H_6_ in Supplementary Figure S5 (ESI†). Not surprisingly, benzene has a π bifurcation value that is close to 1.0. Boroxine and boronyl boroxine (Li et al., 2013) have smaller and yet comparable π bifurcation values (0.64 versus 0.67). The GM B_5_O_6_^−^ (1) cluster has an even smaller π bifurcation value of 0.53, which is marginally π aromatic. The primary reason is that cluster 1 is asymmetrically coordinated so that its hexagonal ring has uneven B−O bond distances (Figure 3).

### A Plausible Mechanism for Structural Transition in the B_5_O_6_^−^ Cluster: From Tetrahedral Isomer to Hexagonal Global Minimum

**FIGURE 10:**
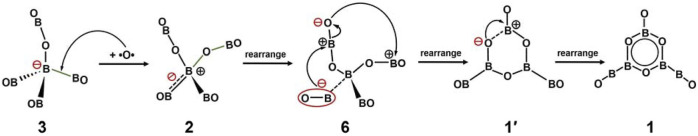
Plausible mechanism for the structural evolution between tetrahedral B_5_O_5_^−^ (3) and hexagonal B_5_O_6_^−^ (1) clusters.

A possible mechanism for tetrahedral-to-hexagonal structure transition from 3/2 to 1 is illustrated in Figure 10. This is an oxygen diradical nucleophilic substitution mechanism. Our starting point is tetrahedral GM B_5_O_5_^−^ (3) cluster, which is substantially more stable than its hexagonal isomer 4 (Figure 1) and inherits the geometry from GM *T*_*d*_ B_5_O_4_^−^ (5) cluster. Upon insertion of an O atom (assumed as oxygen diradical) between the B^−^ center and one boronyl terminal, the negative charge on central B^−^ site would migrate to its surrounding, thus leading to structure 2 (step 1), which is illustrated approximately only. Next, the negatively charged BO migrates to bind a neighboring positively charged B center in the OBO ligand and the charge further migrates so that the former OBO units are ready to fuse (step 2). In other words, the electron-rich boronyl group acts as a nucleophilic reagent to attack a weaker B**–**O link. A key structure 6 is present here. Interestingly, structure 6 has been identified as well in our structural searches, which is the 9th isomer in Figure 2.

Once structure 6 is reached, the next are downhill processes. The negative terminal O center binds with the positive B center in the second OBO ligand and a heterocyclic hexagon is formed (structure 1**′**; step 3). The formation of π sextet from 1**′** results in the final structure of cluster 1 (step 4). The abovementioned mechanism is primarily based on regioselectivity and stereoselectivity (Ren et al., 2017).

The proposed mechanism is reasonable also for a different reason, as outlined in Section *Bond Distances, Wiberg Bond Indices, and Natural Atomic Charges*. Basically, isomer B_5_O_6_^−^ (2) is not an ideal system, despite its tetrahedral configuration similar to those of B_5_O_4_^−^ (5) and B_5_O_5_^−^ (3) (see Figure 4). Ideally, the central site of a tetrahedral molecular system should be valence four so that four Lewis-type single σ bonds can form, such as in CH_4_ and BH_4_^−^. The central B site in B_5_O_4_^−^ (**5**) and B_5_O_5_^−^ (**3**) are in the B^−1.63^ and B^−0.66^ charge states, respectively (Figure 4). In stark contrast, the central B site in B_5_O_6_^−^ (2) is practically neutral (+0.12 |e|) and inconsistent with a formal B^−^ center. As a consequence, the tetrahedral coordination is not favorable for the B_5_O_6_^−^ cluster, which is susceptible to structural transformation.

## Conclusion

The structural and bonding properties of a boron oxide B_5_O_6_^−^ cluster has been studied using computer global structure searches and quantum chemical calculations, revealing a perfectly planar *C*_2*v*_ (^1^A_1_) global minimum geometry. It has a heterocyclic B_3_O_3_ hexagon as the core, which is terminally bonded to two boronyls and one O^−^ ligand, marking the exact onset of a hexagonal ring in the B_5_O_*n*_^−^ (*n* = 1–6) cluster series. The electronic structure shows a rather high vertical electron affinity of greater than 5 eV, suggesting that the species belongs to superhalogen anions. Chemically, the B_5_O_6_^−^ *C*_2*v*_ (^1^A_1_) cluster is a close boronyl-based analog to phenolate anion, in which a boronyl ligand is isovalent to hydrogen and a heteroatomic B_3_O_3_ ring substitutes a C_6_ ring. The two kinds of hexagonal rings are, in effect, isoelectronic with each other in terms of the available number of electrons for bonding within the rings. The B_5_O_6_^−^ *C*_2*v*_ (^1^A_1_) cluster features a π sextet, similar to those in boroxine and boronyl boroxine, thus also rendering the cluster a new member of the “inorganic benzene” family. A plausible mechanism is proposed to account for the tetrahedral-to-hexagonal structure transition in the B_5_O_*n*_^−^ (*n* = 1–6) clusters.

## Data Availability

The original contributions presented in the study are included in the article/Supplementary Material; further inquiries can be directed to the corresponding authors.

## References

[B1] Alexandrova A. N. Boldyrev A. I. Zhai H.-J. Wang L.-S. (2006). All-boron Aromatic Clusters as Potential New Inorganic Ligands and Building Blocks in Chemistry. Coord. Chem. Rev. 250, 2811–2866. 10.1016/j.ccr.2006.03.032

[B2] A Technical Note, Electron Affinity (EA) characterizes the energetic gain of a neutral molecular system upon attachment of an extra charge, that is, its oxidation capability. Among atoms in the periodic table, halogens have the largest EAs (3.06−3.61 eV). In particular, the Cl atom has the greatest value of 3.61 eV. Due to collective effects, certain molecules or polyatomic radicals can possess EAs that are even higher than those of halogens and such exotic systems have been called “superhalogens”. Their corresponding anions are electronically robust, similar to halogen anions. These cluster or molecular anions are therefore called the “superhalogen anions”.

[B3] Bauernschmitt R. Ahlrichs R. (1996). Treatment of Electronic Excitations within the Adiabatic Approximation of Time Dependent Density Functional Theory. Chem. Phys. Lett. 256, 454–464. 10.1016/0009-2614(96)00440-x

[B4] Braunschweig H. Radacki K. Schneider A. (2010). Oxoboryl Complexes: Boron−Oxygen Triple Bonds Stabilized in the Coordination Sphere of Platinum. Science 328, 345–347. 10.1126/science.1186028 20395506

[B5] Casida M. E. Jamorski C. Casida K. C. Salahub D. R. (1998). Molecular Excitation Energies to High-Lying Bound States from Time-dependent Density-Functional Response Theory: Characterization and Correction of the Time-dependent Local Density Approximation Ionization Threshold. J. Chem. Phys. 108, 4439–4449. 10.1063/1.475855

[B6] Chen Q. Bai H. Zhai H.-J. Li S.-D. Wang L.-S. (2013). Photoelectron Spectroscopy of boron-gold alloy Clusters and boron Boronyl Clusters: B_3_Au_ *n* _ ^−^ and B_3_(BO)_ *n* _ ^−^ (*n* = 1, 2). J. Chem. Phys. 139, 044308. 10.1063/1.4816010 23901981

[B7] Chen Q. Lu H.G Zhai H.-J. Li S.-D. (2014). Chemical bonding in electron-deficient boron oxide clusters: core boronyl groups, dual 3c-4e hypervalent bonds, and rhombic 4c-4e bonds. Phys. Chem. Chem. Phys. 16, 7274. 10.1039/c4cp00406j 24619010

[B8] Chen Q. Zhai H.-J. Li S.-D. Wang L.-S. (2012). Probing the Structures and Chemical Bonding of boron-boronyl Clusters Using Photoelectron Spectroscopy and Computational Chemistry: B_4_(BO)_ *n* _ ^−^ (*n* = 1–3). J. Chem. Phys. 137, 044307. 10.1063/1.4737863 22852618

[B9] Doyle R. J. (1988). High-molecular-weight boron Oxides in the Gas Phase. J. Am. Chem. Soc. 110, 4120–4126. 10.1021/ja00221a004

[B10] Drummond M. L. Meunier V. Sumpter B. G. (2007). Structure and Stability of Small Boron and Boron Oxide Clusters. J. Phys. Chem. A. 111, 6539–6551. 10.1021/jp0726182 17583331

[B11] Fagiani M. R. Song X. Petkov P. Debnath S. Gewinner S. Schöllkopf W. (2017). Structure and Fluxionality of B_13_ ^+^ Probed by Infrared Photodissociation Spectroscopy. Angew. Chem. Int. Ed. 56, 501–504. 10.1002/anie.201609766 27918141

[B12] Feng L.-Y. Li R. Zhai H.-J. (2019). Boron-based Inorganic Heterocyclic Clusters: Electronic Structure, Chemical Bonding, Aromaticity, and Analogy to Hydrocarbons. Phys. Chem. Chem. Phys. 21, 20523–20537. 10.1039/c9cp03254a 31304948

[B13] Frisch M. J., et al. (2009). GAUSSIAN 09, Revision D.01. Wallingford, CT: Gaussian, Inc..

[B14] Glendening E. D. Badenhoop J. K. Reed A. E. Carpenter J. E. Bohmann J. A. Morales C. M. (2013). NBO 6.0. Madison: Theoretical Chemistry Institute, University of Wisconsin.

[B15] Guo J.-C. Feng L.-Y. Dong C. Zhai H.-J. (2020). A Designer 32-electron Superatomic CBe_8_H_12_ Cluster: Core-Shell Geometry, Octacoordinate Carbon, and Cubic Aromaticity. New J. Chem. 44, 7286–7292. 10.1039/d0nj00778a

[B16] Guo J.-C. Feng L.-Y. Wang Y.-J. Jalife S. Vásquez-Espinal A. Cabellos J. L. (2017). Coaxial Triple-Layered versus Helical Be_6_B_11_− Clusters: Dual Structural Fluxionality and Multifold Aromaticity. Angew. Chem. Int. Ed. 56, 10174–10177. 10.1002/anie.201703979 28688126

[B17] Guo J.-C. Lu H.-G. Zhai H.-J. Li S.-D. (2013). Face-Capping μ^3^-BO in B_6_(BO)_7_ ^–^: Boron Oxide Analogue of B_6_H_7_ ^–^ with Rhombic 4c-2e Bonds. J. Phys. Chem. A. 117, 11587–11591. 10.1021/jp4089723 24147988

[B18] Gutsev G. L. Boldyrev A. I. (1984). The Way to Systems with the Highest Possible Electron Affinity. Chem. Phys. Lett. 108, 250–254. 10.1016/0009-2614(84)87059-1

[B19] Kandalam A. K. Kiran B. Jena P. Pietsch S. Ganteför G. (2015). Superhalogens Beget Superhalogens: a Case Study of (BO_2_)*n* Oligomers. Phys. Chem. Chem. Phys. 17, 26589–26593. 10.1039/c5cp04600a 26394536

[B20] Li D.-Z. Bai H. Chen Q. Lu H. Zhai H.-J. Li S.-D. (2013). Perfectly Planar Boronyl Boroxine D_3_ *h* B_6_O_6_: A boron Oxide Analog of Boroxine and Benzene. J. Chem. Phys. 138, 244304. 10.1063/1.4811330 23822241

[B21] Li D.-Z. Feng L.-Y. Zhang L.-J. Pei L. Tian W.-J. Li P.-F. (2018). Planar Tricyclic B_8_O_8_ and B_8_O_8_ ^–^ Clusters: Boron Oxide Analogues of *s*-Indacene C_12_H_8_ . J. Phys. Chem. A. 122, 2297–2306. 10.1021/acs.jpca.7b12479 29401396

[B22] Li D. Z. Feng L. Y. Pei L. Song M. Z. Zhang L. J. Wang H. (2019). Structures and Bonding of B_4_O_5_ and B_4_O_5_ ^−^ Clusters: Emergence of Boroxol Ring and Competition Between Rhombic B_2_O_2_ and Hexagonal B_3_O_3_ Cores. Int. J. Quan. Chem. 119, 25907. 10.1002/qua.25907

[B23] Li S.-D. Zhai H.-J. Wang L.-S. (2008). B_2_(BO)_2_ ^2–^ Diboronyl Diborene: A Linear Molecule with a Triple Boron−Boron Bond. J. Am. Chem. Soc. 130, 2573–2579. 10.1021/ja0771080 18251470

[B24] Lu T. Chen F.W. (2012). Multiwfn: A Multifunctional Wavefunction Analyzer. J. Comput. Chem. 33, 580–592. 10.1002/jcc.22885 22162017

[B25] Miao C.-Q. Lu H.-G. Li S.-D. (2013). Covalent Bonding in Au(BO)_2_ ^−^ and Au(BS)_2_ ^−^ . J. Clust. Sci. 24, 233–241. 10.1007/s10876-012-0546-z

[B26] Peiris D. Lapicki A. Anderson S. L. Napora R. Linder D. Page M. (1997). Boron Oxide Oligomer Collision-Induced Dissociation: Thermochemistry, Structure, and Implications for Boron Combustion. J. Phys. Chem. A. 101, 9935–9941. 10.1021/jp972157s

[B27] Purvis G. D. Bartlett R. J. (1982). A Full Coupled‐cluster Singles and Doubles Model: The Inclusion of Disconnected Triples. J. Chem. Phys. 76, 1910–1918. 10.1063/1.443164

[B28] Pyykkö P. Atsumi M. (2009). Molecular Single-Bond Covalent Radii for Elements 1-118. Chem. Eur. J. 15, 12770. 10.1002/chem.200800987 19058281

[B29] Reed A. E. Curtiss L. A. Weinhold F. (1988). Intermolecular Interactions from a Natural Bond Orbital, Donor-Acceptor Viewpoint. Chem. Rev. 88, 899–926. 10.1021/cr00088a005

[B30] Ren S.-C. Zhang F.-L. Qi J. Huang Y.-S. Xu A.-Q. Yan H.-Y. (2017). Radical Borylation/Cyclization Cascade of 1,6-Enynes for the Synthesis of Boron-Handled Hetero- and Carbocycles. J. Am. Chem. Soc. 139, 6050–6053. 10.1021/jacs.7b01889 28402108

[B31] Saunders M. (2004). Stochastic Search for Isomers on a Quantum Mechanical Surface. J. Comput. Chem. 25, 621–626. 10.1002/jcc.10407 14978704

[B32] Schleyer P. v. R. Maerker C. Dransfeld A. Jiao H. van Eikema Hommes N. J. R. (1996). Nucleus-Independent Chemical Shifts: A Simple and Efficient Aromaticity Probe. J. Am. Chem. Soc. 118, 6317–6318. 10.1021/ja960582d 28872872

[B33] Sergeeva A. P. Averkiev B. B. Zhai H.-J. Boldyrev A. I. Wang L.-S. (2011). All-boron Analogues of Aromatic Hydrocarbons: B_17_ ^−^ and B_18_ ^−^ . J. Chem. Phys. 134, 224304. 10.1063/1.3599452 21682511

[B34] Shao C.-B. Jin L. Fu L.-J. Ding Y.-H. (2009). Theoretical Study of B_3_O Radical Isomers and Their Interconversion Pathways. Mol. Phys. 107, 2395–2402. 10.1080/00268970903317247

[B35] Silvi B. Savin A. (1994). Classification of Chemical Bonds Based on Topological Analysis of Electron Localization Functions. Nature 371, 683–686. 10.1038/371683a0

[B36] Srivastava A. K. (2022). M(BO)_ *k*+1_ ^–^ Anions: Novel Superhalogens Based on Boronyl Ligands. J. Phys. Chem. A. 126, 513–520. 10.1021/acs.jpca.1c08773 35077171

[B37] Tai T. B. Nguyen M. T. Dixon D. A. (2010). Thermochemical Properties and Electronic Structure of Boron Oxides B_ *n* _O_ *m* _ (*n* = 5−10, *m* = 1−2) and Their Anions. J. Phys. Chem. A. 114, 2893–2912. 10.1021/jp909512m 20112902

[B38] Tai T. B. Nguyen M. T. (2009). Structure and Electron Delocalization of the boron Oxide Cluster B_3_(BO)_3_ and its Anion and Dianion. Chem. Phys. Lett. 483, 35–42. 10.1016/j.cplett.2009.10.054

[B39] Tian W.-J. You X.-R. Li D.-Z. Ou T. Chen Q. Zhai H.-J. (2015). A First-Principles Study on the B_5_O_5_ ^+/0^ and B_5_O_5_ ^−^ Clusters: The boron Oxide Analogs of C_6_H_5_ ^+/0^ and CH_3_Cl. J. Chem. Phys. 143, 064303. 10.1063/1.4928282 26277134

[B40] Tian W.-J. Zhao L.-J. Chen Q. Ou T. Xu H.-G. Zheng W.-J. (2015). Photoelectron spectroscopy of B_4_O_4_ ^−^: Dual 3c-4e π hyperbonds and rhombic 4c-4e *o*-bond in boron oxide clusters. J. Chem. Phys. 142, 134305. 10.1063/1.4916386 25854241

[B41] von Niessen W. Schirmer J. Cederbaum L. S. (1984). Computational Methods for the One-Particle green’s Function. Comp. Phys. Rep. 1, 57–125. 10.1016/0167-7977(84)90002-9

[B42] Wang Y.-J. Guo J.-C. Zhai H.-J. (2017). Why Nanoscale Tank Treads Move? Structures, Chemical Bonding, and Molecular Dynamics of a Doped boron Cluster B_10_C. Nanoscale 9, 9310–9316. 10.1039/c7nr03193a 28678260

[B43] Yao W.-Z. Guo J.-C. Lu H.-G. Li S.-D. (2009). T_d_ B(BO)_4_ ^−^: A Tetrahedral Boron Oxide Cluster Analogous to Boron Hydride T_d_ BH_4_ ^−^ . J. Phys. Chem. A. 113, 2561–2564. 10.1021/jp809463j 19226113

[B44] Zakrzewski V. G. Ortiz J. V. (1995). Semidirect Algorithms for Third-Order Electron Propagator Calculations. Int. J. Quan. Chem. 53, 583–590. 10.1002/qua.560530602

[B45] Zakrzewski V. G. von Niessen W. (1993). Vectorizable Algorithm for green Function and many-body Perturbation Methods. J. Comput. Chem. 14, 13–18. 10.1002/jcc.540140105

[B46] Zhai H.-J. Chen Q. Bai H. Li S.-D. Wang L.-S. (2014). Boronyl Chemistry: The BO Group as a New Ligand in Gas-phase Clusters and Synthetic Compounds. Acc. Chem. Res. 47, 2435–2445. 10.1021/ar500136j 24915198

[B47] Zhai H.-J. Guo J.-C. Li S.-D. Wang L.-S. (2011). Bridging η^2^-BO in B_2_(BO)_3_ ^−^ and B_3_(BO)_3_ ^−^ Clusters: Boronyl Analogs of Boranes. ChemPhysChem 12, 2549–2553. 10.1002/cphc.201100553 21954002

[B48] Zhai H.-J. Kiran B. Li J. Wang L.-S. (2003). Hydrocarbon Analogues of boron Clusters - Planarity, Aromaticity and Antiaromaticity. Nat. Mater 2, 827–833. 10.1038/nmat1012 14608377

[B49] Zhai H.-J. Li S.-D. Wang L.-S. (2007). Boronyls as Key Structural Units in Boron Oxide Clusters: B(BO)_2_ ^–^ and B(BO)_3_ ^–^ . J. Am. Chem. Soc. 129, 9254–9255. 10.1021/ja072611y 17622148

[B50] Zhai H.-J. Wang L.-M. Li S.-D. Wang L.-S. (2007). Vibrationally Resolved Photoelectron Spectroscopy of BO^–^ and BO_2_ ^–^: A Joint Experimental and Theoretical Study. J. Phys. Chem. A. 111, 1030–1035. 10.1021/jp0666939 17253668

[B51] Zhai H.-J. Wang L.-S. Alexandrova A. N. Boldyrev A. I. (2002). Electronic Structure and Chemical Bonding of B_5_ ^−^ and B_5_ by Photoelectron Spectroscopy Andab Initiocalculations. J. Chem. Phys. 117, 7917–7924. 10.1063/1.1511184

[B52] Zhai H.-J. Zhao Y.-F. Li W.-L. Chen Q. Bai H. Hu H.-S. (2014). Observation of an All-boron Fullerene. Nat. Chem 6, 727–731. 10.1038/nchem.1999 25054944

[B53] Zhao L.-J. Tian W.-J. Ou T. Xu H.-G. Feng G. Xu X.-L. (2016). Structures and Chemical Bonding of B_3_O_3_ ^−/0^ and B_3_O_3_H^−/0^: A Combined Photoelectron Spectroscopy and First-Principles Theory Study. J. Chem. Phys. 144, 124301. 10.1063/1.4943768 27036442

[B54] Zubarev D. Y. Boldyrev A. I. (2008). Developing Paradigms of Chemical Bonding: Adaptive Natural Density Partitioning. Phys. Chem. Chem. Phys. 10, 5207. 10.1039/b804083d 18728862

