# Supplementary material for: Boron Oxide B5O6 − Cluster as a Boronyl-Based Inorganic Analog of Phenolate Anion
Source: Front Chem. 2022 Apr 8;10:868782. doi: 10.3389/fchem.2022.868782 (PMC9024314; doi:10.3389/fchem.2022.868782)

SUPPORTING INFORMATION

Boron oxide $B_5O_6^-$ cluster as a boronyl-based inorganic analog of phenolate anion

Shu-Juan Gao,[a,b] Jin-Chang Guo,*[a] and Hua-Jin Zhai*[a]

[a]*Nanocluster Laboratory, Institute of Molecular Science, Shanxi University, Taiyuan 030006, China*

[b]*Department of Chemistry and Chemical Engineering, Lvliang University, Lvliang 033000, China*

*E-mail: guojc@sxu.edu.cn; hj.zhai@sxu.edu.cn

Full citation of ref. 48.

Table S1. Cartesian coordinates (in Å) for C_{2v} $B_5O_6^-$ (**1**) and C_s $B_5O_6^-$ (**2**) clusters at the PBE0/6-311+G(d) level.

Table S2. Vertical detachment energies (VDEs; in eV) of C_{2v} $B_5O_6^-$ (**1**, $^1A_1$), C_s $B_5O_6^-$ (**2**, $^1A'$), C_s $B_5O_5^-$ (**3**, $^1A'$), and C_s $B_5O_5^-$ (**4**, $^1A'$) clusters. The VDEs have been calculated at the time-dependent PBE0/6-311+G(d) (TD-PBE0), the single-point CCSD(T), and the outer valence Green's function (OVGF) levels, respectively.

Figure S1. Optimized structures of C_s $B_5O_5^-$ (**3**, $^1A'$), C_s $B_5O_5^-$ (**4**, $^1A'$), and T_d $B_5O_4^-$ (**5**, $^1A_1$) clusters at the PBE0/6-311+G(d) level. Bond distances (in Å) are shown in black

color and Wiberg bond indices (WBIs) in blue color. The B atoms are illustrated in blue and O in red.

Figure S2. Optimized bond distances (in Å; black color) of hexagonal C_{2v} ($^2B_2$) versus tetrahedral C_s ($^2A''$) structures of neutral B_5O_6 cluster, at the PBE0/6-311+G(d) level. Also shown are their WBIs (in blue color) from the natural bond orbital (NBO) analysis. The B atoms are shown in blue and O in red.

Figure S3. Calculated natural atomic charges (in |e|) for hexagonal C_{2v} ($^2B_2$) versus tetrahedral C_s ($^2A''$) structures of neutral B_5O_6 cluster from the NBO analysis. The B atoms are shown in blue and O in red.

Figure S4. Selected canonical molecular orbitals (CMOs) of (a) $B_5O_6^-$ (**2**) cluster, as compared to those of (b) $B_5O_5^-$ (**3**) cluster (ref. [20]). These CMOs illustrate the concept of dual four-center four-electron (4c-4e) π bonds, both in-plane and out-of-plane, in the vicinity of a terminal OBO unit.

Figure S5. Electron localization function (ELF) for the π framework, that is, ELF_π, in $B_5O_6^-$ (**1**) cluster, as compared to those of the relevant boroxine $B_3O_3H_3$ (D_{3h}, $^1A_1'$), boronyl boroxine B_6O_6 (D_{3h}, $^1A_1'$), and benzene C_6H_6 (D_{6h}, $^1A_{1g}$) systems. The π bifurcation values increase along the series, indicating that $B_5O_6^-$ (**1**) cluster has rather weak π aromaticity despite its formal π sextet framework.

Full citation of ref. 48.

48 *GAUSSIAN 09*, Revision D.01, M. J. Frisch, G. W. Trucks, H. B. Schlegel, G. E. Scuseria, M. A. Robb, J. R. Cheeseman, G. Scalmani, V. Barone, B. Mennucci, G. A. Petersson, H. Nakatsuji, M. Caricato, X. Li, H. P. Hratchian, A. F. Izmaylov, J. Bloino, G. Zheng, J. L. Sonnenberg, M. Hada, M. Ehara, K. Toyota, R. Fukuda, J. Hasegawa, M. Ishida, T. Nakajima, Y. Honda, O. Kitao, H. Nakai, T. Vreven, J. A. Montgomery, Jr., J. E. Peralta, F. Ogliaro, M. Bearpark, J. J. Heyd, E. Brothers, K. N. Kudin, V. N. Staroverov, R. Kobayashi, J. Normand, K. Raghavachari, A. Rendell, J. C. Burant, S. S. Iyengar, J. Tomasi, M. Cossi, N. Rega, J. M. Millam, M. Klene, J. E. Knox, J. B. Cross, V. Bakken, C. Adamo, J. Jaramillo, R. Gomperts, R. E. Stratmann, O. Yazyev, A. J. Austin, R. Cammi, C. Pomelli, J. W. Ochterski, R. L. Martin, K. Morokuma, V. G. Zakrzewski, G. A. Voth, P. Salvador, J. J. Dannenberg, S. Dapprich, A. D. Daniels, Ö. Farkas, J. B. Foresman, J. V. Ortiz, J. Cioslowski, and D. J. Fox, Gaussian, Inc., Wallingford CT, 2009.

Table S1. Cartesian coordinates (in Å) for C_{2v} $B_5O_6^-$ (**1**) and C_s $B_5O_6^-$ (**2**) clusters at the PBE0/6-311+G(d) level.

$B_5O_6^-$ (**1**, C_{2v}, $^1A_1$)

B	0.00000000	1.20660600	-0.3075550
B	0.00000000	-2.6436820	-1.1705360
B	0.00000000	-1.2066060	-0.3075550
B	0.00000000	0.00000000	1.93908200
B	0.00000000	2.64368200	-1.1705360
O	0.00000000	-3.7016170	-1.8075110
O	0.00000000	-1.2010660	1.04005300
O	0.00000000	1.20106600	1.04005300
O	0.00000000	0.00000000	3.20995000
O	0.00000000	0.00000000	-1.0393470
O	0.00000000	3.70161700	-1.8075110

$B_5O_6^-$ (**2**, C_s, $^1A'$)

B	-2.1792890	0.51189000	0.00000000
B	0.27854200	-1.3263640	1.40274700
B	0.27590000	-0.3332960	0.00000000
B	1.71373200	1.77162400	0.00000000
B	0.27854200	-1.3263640	-1.40274700
O	0.88954600	0.60317100	0.00000000
O	0.27854200	-2.0525710	-2.37112800
O	3.40225200	0.48415400	0.00000000
O	1.51220500	0.49331800	0.00000000

O	1.99286800	2.96356900	0.00000000
O	0.27854200	-2.0525710	2.37112800

Table S2. Vertical detachment energies (VDEs; in eV) of C_{2v} $B_5O_6^-$ (**1**, $^1A_1$), C_s $B_5O_6^-$ (**2**, $^1A'$), C_s $B_5O_5^-$ (**3**, $^1A'$), and C_s $B_5O_5^-$ (**4**, $^1A'$) clusters. The VDEs have been calculated at the time-dependent PBE0/6-311+G(d) (TD-PBE0), the single-point CCSD(T), and the outer valence Green's function (OVGF) levels, respectively.

					TD-PBE0	CCSD(T)	OVGF
species	transition	CMO	feature	final state	VDE[a]	VDE[a]	VDE[a]
**1**	$^2B_2 \leftarrow {}^1A_1$	HOMO (b_2)	X	$^2B_2$	**5.26**	**5.14**	5.91
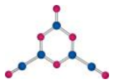	$^2A_2 \leftarrow {}^1A_1$	HOMO–1 (b_1)	A	$^2A_2$	**6.09**		
	$^2A_1 \leftarrow {}^1A_1$	HOMO–2 (b_2)	B	$^2A_1$	**6.93**		
**2**	$^2B_2 \leftarrow {}^1A'$	HOMO (b_2)	X	$^2B_2$	**6.47**	**6.57**	6.72
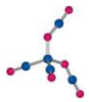	$^2A'' \leftarrow {}^1A'$	HOMO–1 (b_1)	A	$^2A''$	**7.51**		
	$^2A'' \leftarrow {}^1A'$	HOMO–2 (a_1)	B	$^2A''$	**7.60**		
**3**	$^2A'' \leftarrow {}^1A'$	HOMO (a_1)	X	$^2A''$	**6.85**	**6.97**	7.07
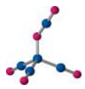	$^2A'' \leftarrow {}^1A'$	HOMO–1 (b_2)	A	$^2A''$	**7.09**		
	$^2A'' \leftarrow {}^1A'$	HOMO–2 (a_1)	B	$^2A''$	**7.83**		
**4**	$^2A' \leftarrow {}^1A'$	HOMO (a′)	X	$^2A'$	**2.26**	**2.27**	2.47
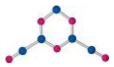	$^2A' \leftarrow {}^1A'$	HOMO–1 (a″)	A	$^2A'$	**6.33**		
	$^2A'' \leftarrow {}^1A'$	HOMO–2 (a″)	B	$^2A''$	**7.07**		

[a] The three levels of theory can be calibrated using a prior $B_4O_3^-$ (D_{3h}, $^1A_1'$) cluster, which has been experimentally characterized via anion photoelectron spectroscopy (ref. [9]). Our calculated ground-state VDE values are 3.88, 3.40, and 3.18 eV, respectively, at the time-dependent PBE0/6-311+G(d) (TD-PBE0), single-point CCSD(T)/6-311+G(d) (on the basis of optimized PBE0-D3/6-311+G(d) geometry), and OVGF/6-311+G(d) levels. The values are to be compared to the experiment data of 3.64 ± 0.03 eV. Thus, OVGF seems to be of poor performance. The PBE0 and CCSD(T) methods are relatively more reliable in terms of electron binding energies.

Figure S1. Optimized structures of C_s $B_5O_5^-$ (**3**, $^1A'$), C_s $B_5O_5^-$ (**4**, $^1A'$), and T_d $B_5O_4^-$ (**5**, $^1A_1$) clusters at the PBE0/6-311+G(d) level. Bond distances (in Å) are shown in black color and Wiberg bond indices (WBIs) in blue color. The B atoms are illustrated in blue and O in red.

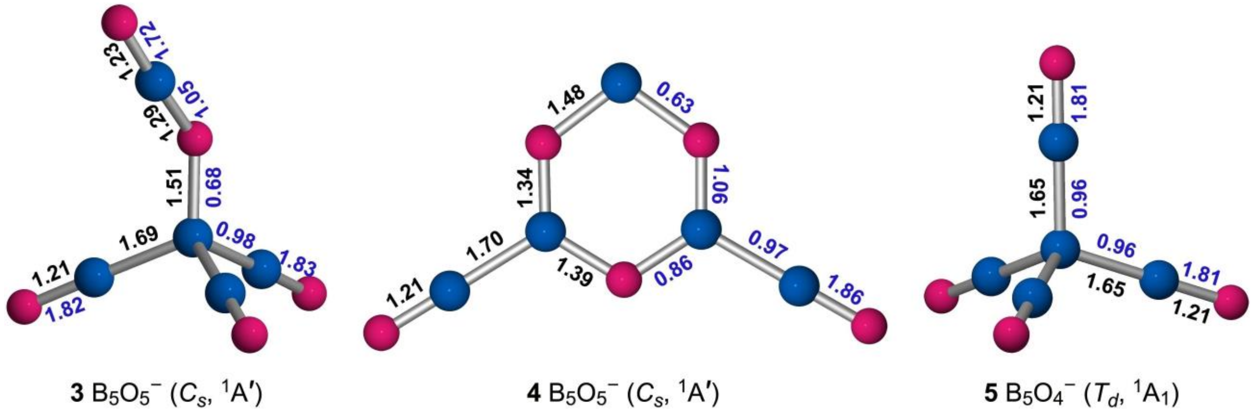

Figure S2. Optimized bond distances (in Å; black color) of hexagonal C_{2v} ($^2B_2$) versus tetrahedral C_s ($^2A''$) structures of neutral B_5O_6 cluster, at the PBE0/6-311+G(d) level. Also shown are their WBIs (in blue color) from the natural bond orbital (NBO) analysis. The B atoms are shown in blue and O in red.

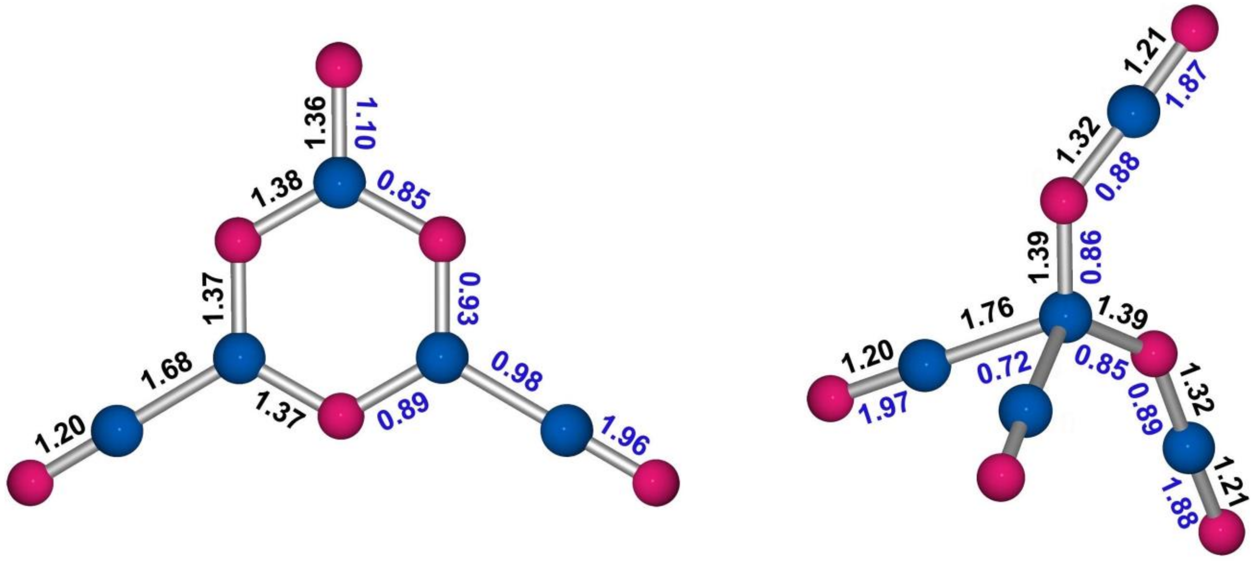

B_5O_6 (C_{2v}, $^2B_2$) B_5O_6 (C_s, $^2A''$)

Figure S3. Calculated natural atomic charges (in |e|) for hexagonal C_{2v} ($^2B_2$) versus tetrahedral C_s ($^2A''$) structures of neutral B_5O_6 cluster from the NBO analysis. The B atoms are shown in blue and O in red.

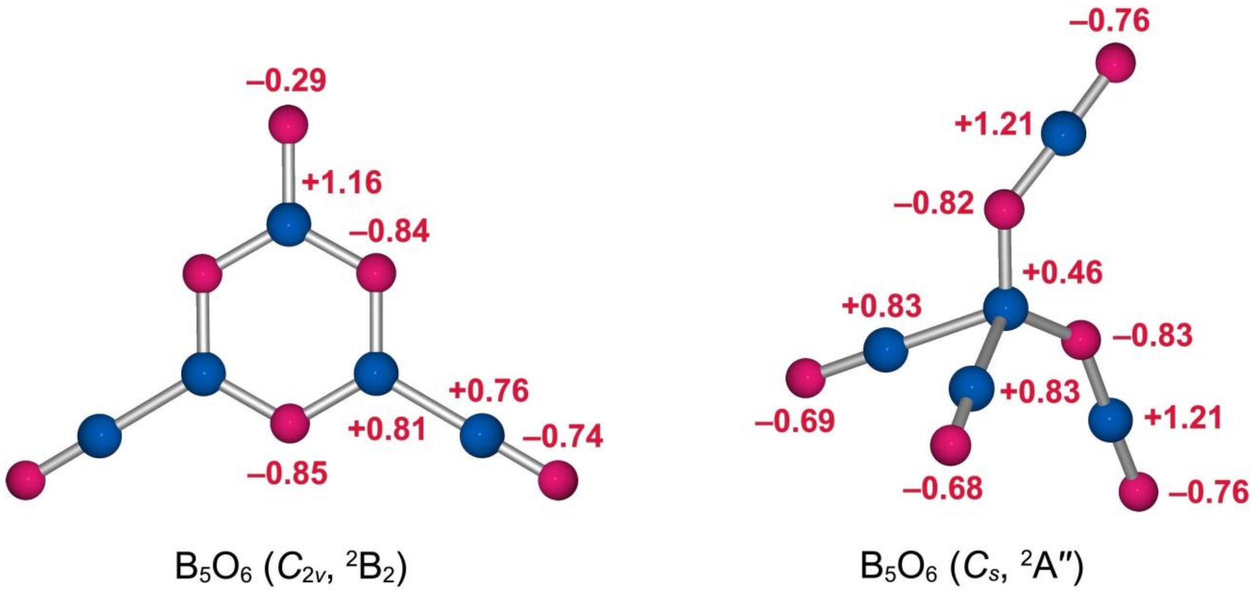

Figure S4. Selected canonical molecular orbitals (CMOs) of (a) $B_5O_6^-$ (**2**) cluster, as compared to those of (b) $B_5O_5^-$ (**3**) cluster (ref. [20]). These CMOs illustrate the concept of dual four-center four-electron (4c-4e) π bonds, both in-plane and out-of-plane, in the vicinity of a terminal OBO unit.

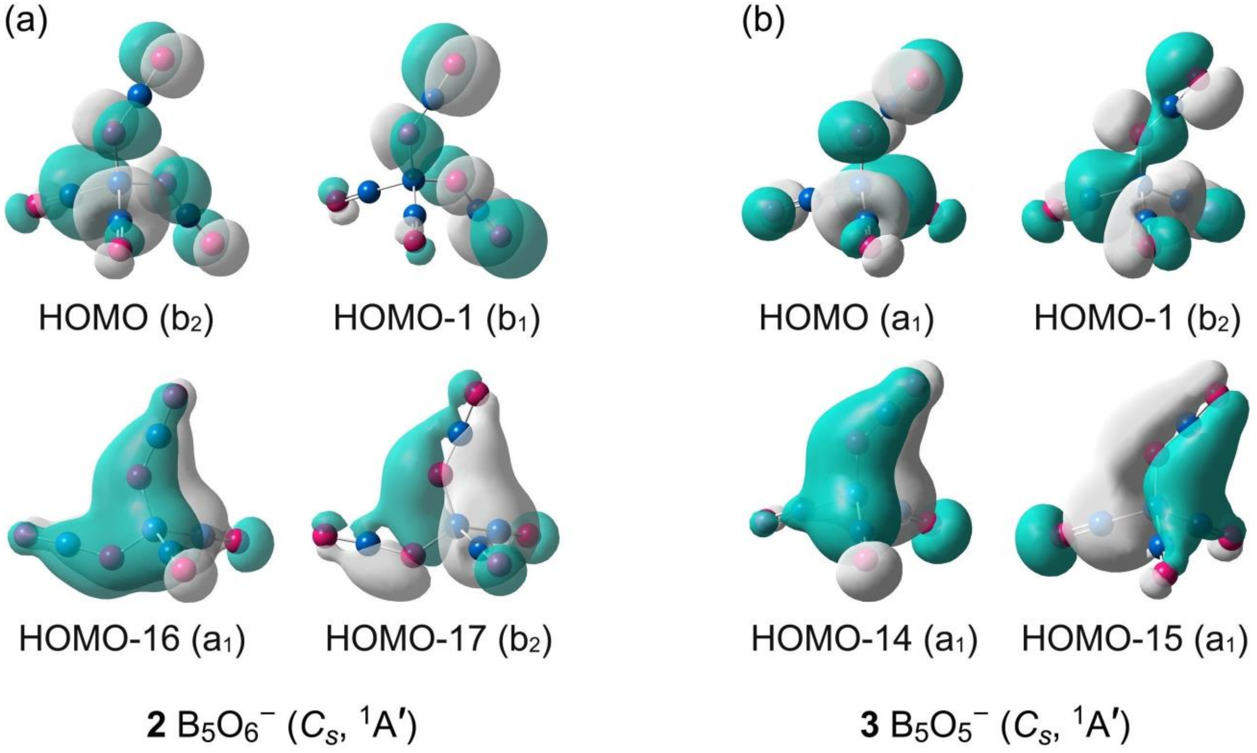

Figure S5. Electron localization function (ELF) for the π framework, that is, ELF_π, in $B_5O_6^-$ (**1**) cluster, as compared to those of the relevant boroxine $B_3O_3H_3$ (D_{3h}, $^1A_1'$), boronyl boroxine B_6O_6 (D_{3h}, $^1A_1'$), and benzene C_6H_6 (D_{6h}, $^1A_{1g}$) systems. The π bifurcation values increase along the series, indicating that $B_5O_6^-$ (**1**) cluster has rather weak π aromaticity despite its formal π sextet framework.

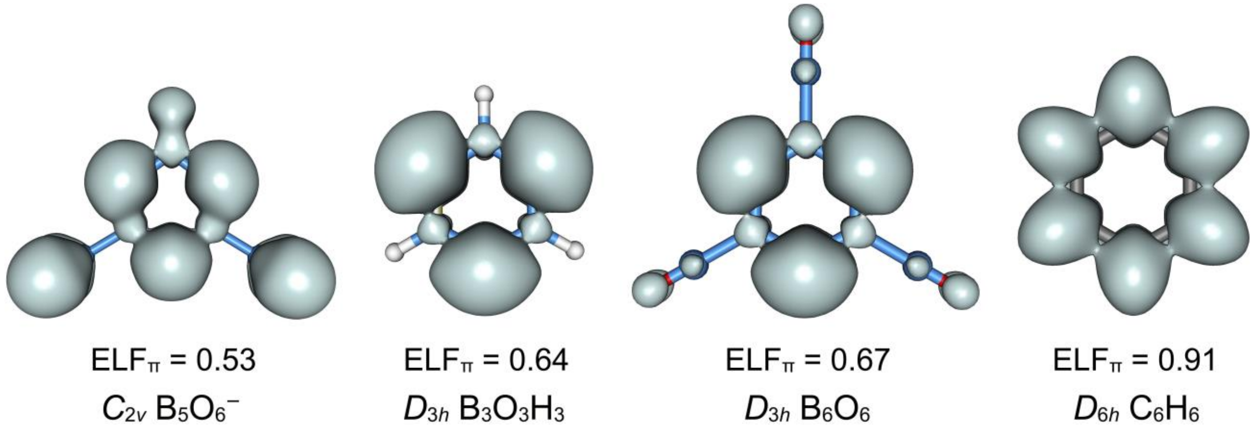

Supplement: Supplementary file 1 [file DataSheet1.pdf]
